# Trends and Risk Factors of In-Hospital Mortality of Patients with COVID-19 in Germany: Results of a Large Nationwide Inpatient Sample

**DOI:** 10.3390/v14020275

**Published:** 2022-01-28

**Authors:** Lukas Hobohm, Ingo Sagoschen, Stefano Barco, Irene Schmidtmann, Christine Espinola-Klein, Stavros Konstantinides, Thomas Münzel, Karsten Keller

**Affiliations:** 1 Department of Cardiology, University Medical Center Mainz (Johannes Gutenberg-University Mainz), 55131 Mainz, Germany; lukas.hobohm@unimedizin-mainz.de (L.H.); Ingo.Sagoschen@unimedizin-mainz.de (I.S.); espinola@uni-mainz.de (C.E.-K.); tmuenzel@uni-mainz.de (T.M.); 2 Center for Thrombosis and Hemostasis (CTH), University Medical Center Mainz (Johannes Gutenberg-University Mainz), 55131 Mainz, Germany; stefano.barco@usz.ch (S.B.); stavros.konstantinides@unimedizin-mainz.de (S.K.); 3 Department of Angiology, University Hospital Zurich, 8091 Zurich, Switzerland; 4 Institute of Medical Biostatistics, Epidemiology and Informatics (IMBEI), University Medical Center Mainz (Johannes Gutenberg-University Mainz), 55131 Mainz, Germany; Irene.Schmidtmann@unimedizin-mainz.de; 5 Department of Cardiology, Democritus University of Thrace, 67100 Alexandroupolis, Greece; 6 German Center for Cardiovascular Research (DZHK), Partner Site Rhine Main, 55131 Mainz, Germany; 7 Medical Clinic VII, Department of Sports Medicine, University Hospital Heidelberg, 69120 Heidelberg, Germany

**Keywords:** COVID-19, SARS-CoV2, health resources, mechanical ventilation, intensive care unit

## Abstract

Unselected data of nationwide studies of hospitalized patients with COVID-19 are still sparse, but these data are of outstanding interest to avoid exceeding hospital capacities and overloading national healthcare systems. Thus, we sought to analyze seasonal/regional trends, predictors of in-hospital case-fatality, and mechanical ventilation (MV) in patients with COVID-19 in Germany. We used the German nationwide inpatient samples to analyze all hospitalized patients with a confirmed COVID-19 diagnosis in Germany between 1 January and 31 December in 2020. We analyzed data of 176,137 hospitalizations of patients with confirmed COVID-19-infection. Among those, 31,607 (17.9%) died, whereby in-hospital case-fatality grew exponentially with age. Overall, age ≥ 70 years (OR 5.91, 95%CI 5.70–6.13, *p* < 0.001), pneumonia (OR 4.58, 95%CI 4.42–4.74, *p* < 0.001) and acute respiratory distress syndrome (OR 8.51, 95%CI 8.12–8.92, *p* < 0.001) were strong predictors of in-hospital death. Most COVID-19 patients were treated in hospitals in urban areas (*n* = 92,971) associated with the lowest case-fatality (17.5%), as compared to hospitals in suburban (18.3%) or rural areas (18.8%). MV demand was highest in November/December 2020 (32.3%, 20.3%) in patients between the 6th and 8th age decade. In the first age decade, 78 of 1861 children (4.2%) with COVID-19-infection were treated with MV, and five of them died (0.3%). The results of our study indicate seasonal and regional variations concerning the number of COVID-19 patients, necessity of MV, and case fatality in Germany. These findings may help to ensure the flexible allocation of intensive care (human) resources, which is essential for managing enormous societal challenges worldwide to avoid overloaded regional healthcare systems.

## 1. Take-Home Message

We identified important seasonal and regional differences concerning the number of COVID-19 patients, the necessity of mechanical ventilation, and case fatality. Most COVID-19 patients were treated in hospitals in urban areas associated with the lowest case fatality, as compared to hospitals in suburban or rural areas, and mechanical ventilation demand was highest in November and December 2020. Age ≥ 70 years, pneumonia, and acute respiratory distress syndrome were strong predictors of in-hospital death. These findings may help to ensure a flexible allocation of intensive care (human) resources, which is essential for managing enormous societal challenges worldwide and avoiding overloaded regional health care systems.

## 2. Introduction

In early December 2019, the first patient-cases of pneumonia caused by a previously unknown virus were identified in China [1,2]. The first SARS-CoV-2 infection in Germany was reported at the end of January 2020 in Bavaria [3]. From this initial cluster of coronavirus disease 2019 (COVID-19), sustained transmission of SARS-CoV-2 resulted in a strong spread of COVID-19 in the German population [4]. The total number of patients necessitating admission to intensive care units (ICU) and mechanical ventilation (MV) must be taken into account for adequate future health care planning [5,6]. In order to restrict the spread of the pathogen, to avoid a critical overload of the healthcare system and particularly of the ICUs, many countries have implemented lockdown strategies [4,5,6]. Since the beginning of the pandemic, worldwide deaths related to COVID-19 surpassed 5 million people and more than 100,000 deaths in Germany [7]. Especially, the case-fatality of COVID-19 patients with MV is very high [5,6]. Thus, the necessity of ICU treatment and the need for MV in COVID-19 patients are considered key factors determining a critical overload of the national healthcare system. The 4th infection wave of SARS-CoV-2 infection caused substantial harm with increasing death rates in Germany and all over Europe despite vaccination of more than two-thirds (68.2% on the 25th of November 2021) of the German population. However, vaccination rates strongly vary by region, with concordantly high incidence rates of COVID-19 infections in regions with a low proportion of vaccination [8]. Nationwide, unbiased, and unselected data of hospitalized patients with COVID-19 are rare but of outstanding importance for optimal health care planning and better and efficient management of these infected patients [6,9]. Based on the knowledge regarding regional differences, substantial epicenters of the COVID-19 pandemic with very high mortality rates and a high burden of patients with a need of MV, it is of utmost importance to identify trends and factors affecting increased morbidity, as well as factors predisposing for MV. Thus, our aim was to provide detailed information about patient characteristics, resource use, and outcomes of hospitalized patients with COVID-19 in Germany, to enable better health care coordination, avoid a bottleneck of hospital capacities and, in particular, shortcomings with respect to ventilated ICU beds availabilities [6].

## 3. Methods

### 3.1. Data Source

Statistical analyses were performed on our behalf by the Research Data Center (RDC) of the Federal Bureau of Statistics (Wiesbaden, Germany). Aggregated statistics were provided from RDC on the basis of our SPSS codes (IBM Corp. Released 2011. IBM SPSS Statistics for Windows, Version 20.0. IBM Corp: Armonk, NY, USA), which we had supplied to the RDC (source: RDC of the Federal Statistical Office and the Statistical Offices of the federal states, DRG Statistics 2020, own calculations) [10,11].

With this data analysis of the German nationwide inpatient sample, we aimed to analyze temporal trends of all hospitalized patients with a confirmed COVID-19 diagnosis (ICD-code U07.1) during the observational period between 1 January and 31 December 2020 and identify independent predictors of MV and in-hospital death.

### 3.2. Study Oversight and Support

Since our study did not comprise direct access by the investigators to individual patient data but only access to summarized results provided by the RDC, approval by an ethics committee as well as patients’ informed consent were not required, in accordance with German law [10,11].

### 3.3. Coding of Diagnoses, Procedures and Definitions

In 2004, a diagnosis- and procedure-related remuneration system was introduced in Germany. Coding according to the German Diagnosis Related Groups (G-DRG) system on patient diagnoses, coexisting conditions, and on surgeries, as well as on procedures/interventions, and transferring these codes to the Institute for the Hospital Remuneration System is mandatory for German hospitals to get their remuneration [10,11]. Patients’ diagnoses are coded according to the International Statistical Classification of Diseases and Related Health Problems, 10th revision, with German modification (ICD-10-GM) [10,11]. In parallel, surgical, diagnostic, and interventional procedures were coded according to OPS codes (Operationen- und Prozedurenschlüssel). With the present analysis of the German nationwide inpatient sample, we were able to identify all hospitalized patients with a confirmed COVID-19 diagnosis (ICD-code U07.1) in Germany since the beginning of the year 2020 (with COVID-19 as the main or secondary diagnosis).

To obtain data regarding coexisting conditions and complications, we used the available diagnostic and procedural codes for acute and chronic conditions (OPS and ICD-10-GM codes), which are presented with related ICD and OPS coding in Table S1 of the Supplementary. Post-COVID was defined as a status of previous survived COVID-19-infection before the patient’s hospitalization with the recurrent COVID-19 infection.

### 3.4. Statistical Analysis

Differences in patient characteristics between the groups of hospitalized COVID-19 patients with MV vs. without MV and COVID-19 patients who died in hospital and those who were discharged alive were calculated with Wilcoxon–Whitney U test for continuous variables and Fisher’s exact or Chi-square test for categorical variables, as appropriate. Temporal trends regarding hospitalizations of COVID-19 patients, use of MV, and in-hospital mortality over time and with increasing age were estimated by means of linear regression analyses. Logistic regression models were calculated to investigate associations between patients’ characteristics, adverse events, and (i) the necessity of MV and (ii) in-hospital death. Results were presented as Odds Ratios (OR) and 95% confidence intervals (CI). All statistical analyses were carried out with the use of SPSS software (IBM Corp. Released 2011. IBM SPSS Statistics for Windows, Version 20.0. IBM Corp: Armonk, New York, NY, USA). Only *p* values of <0.05 (two-sided) were considered to be statistically significant. No adjustment for multiple testing was applied.

## 4. Results

### 4.1. Baseline Characteristics

In total, 176,137 hospitalizations with confirmed COVID-19-infection were coded in Germany during the year 2020. The majority were men (*n* = 92,188; 52.3%) and aged 70 years or older (*n* = 94,329; 53.6%). Cardiovascular comorbidities were common in hospitalized patients with confirmed COVID-19-infections. Overall, almost half of the patients (46.8%; *n* = 82,480) had arterial hypertension and 25,574 (14.4%) were diagnosed with coronary artery disease. In 60.7% (*n* = 106,913) of the hospitalizations, pneumonia was reported, 8.6% (*n* = 15,061) had an acute infection of the upper or lower airways other than pneumonia, and 6.6% (*n* = 11,594) suffered from an acute respiratory distress syndrome (ARDS) during hospitalization (Table 1).

**Figure 1:**
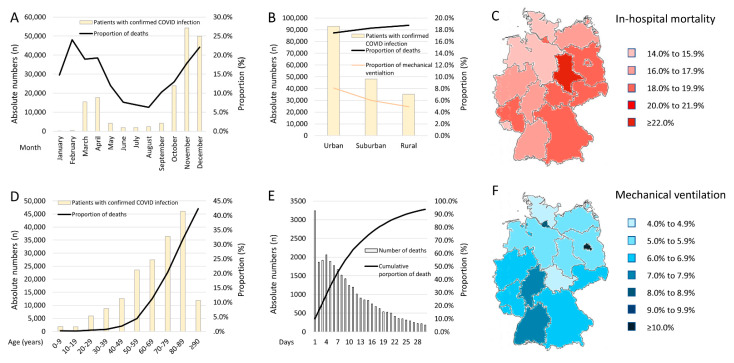
(Facing page). Temporal and regional trends regarding total numbers of hospitalized patients with COVID-19-infection, in-hospital mortality, and mechanical ventilation. Panel (**A**)—Temporal trends regarding total numbers of hospitalized patients with COVID-19-infection (yellow bars) and in-hospital mortality rate (black line) stratified for months. Panel (**B**)—Regional trends regarding total numbers of hospitalized patients with COVID-19-infection (yellow bars), mechanical ventilation (orange line), and in-hospital mortality rate (black line). Panel (**C**)—Regional trends regarding in-hospital death rates of hospitalized patients with COVID-19-infection. Panel (**D**)—Temporal trends regarding total numbers of hospitalized patients with COVID-19-infection (yellow bars) and in-hospital mortality rate (black line) stratified for age decades. Panel (**E**)—Temporal trends regarding deaths of hospitalized patients with COVID-19-infection stratified for days of hospitalization. Panel (**F**)—Regional trends regarding rates of mechanical ventilation of hospitalized patients with COVID-19-infection.

**Figure 2:**
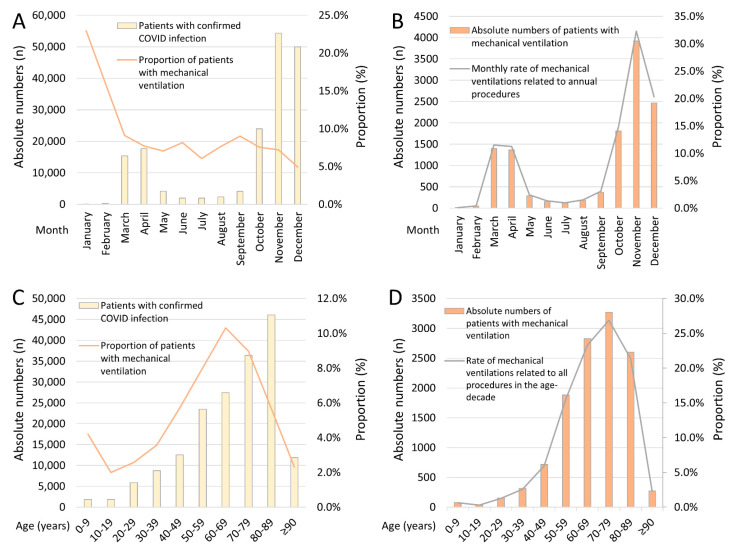
Temporal trends regarding total numbers of hospitalized patients with COVID-19-infection and mechanical ventilation in Germany 2020. Panel (**A**)—Temporal trends regarding total numbers of hospitalized patients with COVID-19-infection (yellow bars), as well as the proportion of COVID-patients with mechanical ventilation (orange line), stratified for months. Panel (**B**)—Temporal trends regarding total numbers of hospitalized COVID-19 patients with mechanical ventilation (orange bars) and percentage of annual mechanic ventilation procedures (grey line) stratified for months. Panel (**C**)—Temporal trends regarding total numbers of hospitalized patients with COVID-19-infection (yellow bars), as well as COVID-patients with mechanical ventilation (orange line), stratified for age decades. Panel (**D**)—Temporal trends regarding total numbers of hospitalized COVID-19 patients with mechanical ventilation (orange bars) and percentage of annual mechanic ventilation procedures (grey line) stratified for age decades.

In total, 31,607 (17.9%) patients with COVID-19 died. More than half of these deaths (50.4%) occurred during the first eight days of hospitalization (Figure 1E). The case-fatality rate increased with patients’ age (β 1.61 per decade, 95% CI, 1.59 to 1.64, *p* < 0.001) with a peak at the 8th and 9th life-decade (Figure 1D). In parallel, the prevalent use of MV was highest in the 6th to 8th life-decade (β 0.06, 95% CI 0.02 to 0.09, *p* = 0.002) (Figure 2C). Remarkably, between the 6th and 8th life-decade, more than one-fifth of the COVID-19 patients required MV (Figure 2C,D). COVID-19 patients with MV were younger and more often males (Table 2). Patients with MV had more often a post-COVID-status than those without (53 [0.4%] vs. 504 [0.3%], *p* = 0.016).

More than 1800 children aged between 0 and 9 years were hospitalized with a COVID-19 infection. Of these, 78 (4.2%) children needed MV (Table 1, Figure 2C), and five of them died (0.3%) during the in-hospital course (Figure 1D).

### 4.2. Comparison of Survivors vs. Non-Survivors in COVID-19 Patients

Non-survivors were older and had approximately 30% higher prevalence of pneumonia and 4.3-fold higher frequency of ARDS (Table 1 and Figure S1 of the Supplementary). Venous thromboembolism (VTE), myocardial infarction, and acute kidney failure occurred more often in deceased patients. Non-survivors were more often treated in the intensive care unit, had higher use of extracorporeal membrane oxygenation (ECMO), MV, and dialysis. Post-COVID-status was more prevalent in survivors (485 [0.3%] vs. 72 [0.2%], *p* = 0.002) (Table 1). The mortality rate increased with the severity of ARDS (Figure S2 of the Supplementary).

### 4.3. Seasonal Trends

In line with an increased number of patients with confirmed COVID-19-infection from January (*n* = 60) to December (*n* = 49,986) 2020, the number of in-hospital deaths increased in parallel from nine patients (14.8%) in January to 11,047 (22.1%) patients in December (Figure 1A). Although the proportion of COVID-19 patients with MV, in relation to the total numbers of patients with COVID-19, decreased over the 1-year observational period (β −0.49 [95%CI −0.55 to −0.43], *p* < 0.001) (Figure 2A,B), the highest proportions of MV among all MVs were reported in November (32.3%) and December (20.3%) (Figure 2B).

### 4.4. Regional Trends of Hospitalized COVID-19 Patients

Most patients with confirmed COVID-19 were treated in hospitals in urban areas (*n* = 92,971) attributed with the lowest case-fatality (17.5%) compared to hospitals in suburban (18.3%) or rural areas (18.8%) (Figure 1B). The lowest case fatality was observed in the federal states of Bremen (14.1%) and Schleswig-Holstein (15.3%), as opposed to Saxony (23.1%) and Hamburg (19.9%) (Figure 1C). The highest use of MV was reported for Berlin (12.1%) and Hamburg (9.9%) (Figure 1F and Figure S3 of the Supplementary).

### 4.5. Predictors of In-Hospital Case-Fatality and Mechanical Ventilation

**Figure 3:**
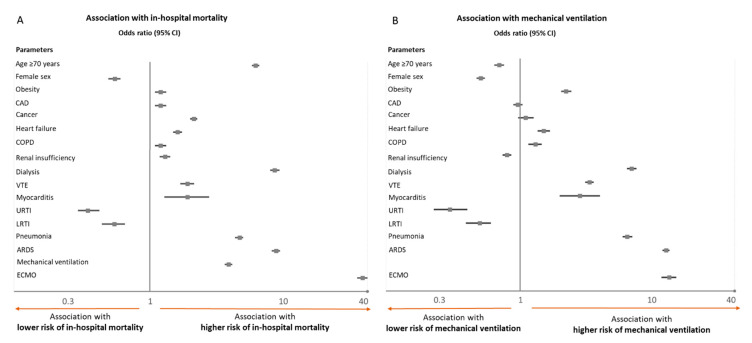
Association of baseline and clinical parameters of COVID-19 patients with in-hospital mortality (**A**) and necessity of mechanical ventilation (**B**). Associations were presented as Odds Ratios (OR) and included the following parameters for adjustment: age, sex, cancer, coronary artery disease, heart failure, COPD, arterial hypertension, renal insufficiency (comprised diagnosis of chronic renal insufficiency stages 3 to 5 with glomerular filtration rate < 60 mL/min/1.73 m^2^), diabetes mellitus, atrial fibrillation, peripheral artery disease, and hyperlipidemia. Abbreviations: CAD, coronary artery disease; COPD, chronic obstructive pulmonary disease; VTE, venous thromboembolism, URTI, upper respiratory tract infection; LRTI, lower respiratory tract infection, other than pneumonia; ARDS, acute respiratory distress syndrome; ECMO, extracorporeal membrane oxygenation.

Several independent predictors of in-hospital case-fatality and MV were detected in a multivariate logistic regression model (Figure 3). Briefly, age ≥ 70 years, dialysis, pneumonia, and ARDS, had a strong association with increased in-hospital death (Figure 3A). If MV (OR 3.82, 95%CI 3.65–4.00, *p* < 0.001) or ECMO (OR 36.78, 95%CI 32.40–41.76, *p* < 0.001) was needed for invasive treatment, the in-hospital case-fatality was substantially increased in COVID-19 patients. In contrast, female sex and non-severe upper or lower respiratory tract infections, other than pneumonia, were associated with a favorable in-hospital course.

The strongest association regarding the risk for MV during the in-hospital course was calculated for patients with obesity, VTE, pneumonia, and ARDS. Associated with a low risk for MV during the in-hospital course were parameters such as female sex, non-severe upper or lower respiratory tract infections other than pneumonia, and age ≥ 70 years (Figure 3B).

## 5. Discussion

The aim of the present study was to examine patient characteristics, use of health care resources, and clinical outcome of more than 176,000 hospitalizations of COVID-19 patients in German hospitals during the year 2020. In comparison, in the United States, a higher number (960,000–2.4 million) of COVID-19–related hospitalizations have been estimated through fall 2020 [7,12]. The 4th wave of the COVID-19-pandemic hits several countries hard, and worldwide deaths related to COVID-19 surpassed 5 million people, including more than 100,000 deaths in Germany until the end of December 2021 [7]. Our analysis showed a U-wave-shaped curve of admissions and death numbers of COVID-19 patients in Germany, with the lowest numbers during the summer season of 2020. Particularly, the reduced in-hospital mortality rate of COVID-19 patients during summer indicates that increased infection rates and, consequently, a higher patient-load at the hospitals, but also important comorbidities in the cold seasons, significantly contribute to higher mortality rates of COVID-19 patients during fall and winter. During January 2021, at the peak of the pandemic in the United States, 79% of the hospital beds at the ICUs were occupied by COVID-19 patients despite a general urge for available ICU capacities for other acute diseases and surgeries [12]. In Europe, the fatality of the pandemic during the first months of 2020 was high, with a case-fatality rate of 8.3% and 250 deaths per one-million citizens [13]. However, significant variations regarding deaths per million infections and the in-hospital case-fatality rate across the European countries were observed [13]. The number of deaths per million citizens was lowest in Slovakia and highest in Belgium, while Germany was located in the middle [13]. The case-fatality rate ranged from 0.5% in Iceland and Belarus to 19.0% in France during the first months of 2020 [13,14], while our data also showed a high case-fatality rate of the hospitalized COVID-19 patients in Germany (17.9%) in the year 2020.

Studies have revealed a high mortality rate of COVID-19 patients admitted to ICUs and emphasized the outstanding importance of an accessible percentage of available ICU beds and ventilators in order to manage and treat all patients admitted to ICU [5]. The aforementioned data demonstrated that more than two-thirds of the overall MV cases in the year 2020 accumulated within the last calendar quarter, implicating the importance of maneuvering freely within the given general ICU capacity range in order to not deprive non-COVID patients of their medical needs.

The 4th wave of the COVID-19-pandemic in Germany has hit hard, causing substantial harm to the health care system, although the majority of the population have been fully vaccinated (at 25th of November 2021, 66.8% of the population) [8]. Several studies have clearly demonstrated that vaccination is a highly effective tool to reduce the spread of symptomatic COVID-19, but also to provide protection against critical illness [15,16,17,18]. Since the vaccination program in Germany did not start before late December 2020, the present study analysis contains vaccination-naive patient data. Despite the progress of the vaccination program in Germany to the current date (approximately 1/3 of the population is not yet two times vaccinated [8]), the unvaccinated minority appears to be the most important accelerator of the 4th infection wave in mid-Europe [19]. Moreover, the waning of immunity, the increased prevalence in the elderly, and more aggressive variants of the virus decreased the effect of vaccinations on the ongoing pandemic [20]. A recent study demonstrated that a third “booster” vaccination jab substantially lowered the likelihood of severe disease [21]. Until November 2021, only 10% of the German population received this third vaccination dose [8]. Besides speeding up the vaccination program and implementing again strict adherence to non-pharmacological public health measures [3,19,20,21], early identification of hospitalized COVID-19 patients, who are at increased risk of severe disease, is crucial to managing healthcare resources.

Our study demonstrates several risk factors of MV as one life-saving escalation of invasive treatment and resource-binding complication as well as in-hospital death. More than 90% of ventilated patients with COVID-19-infection suffered from pneumonia and about 40% of those developed ARDS. Post-COVID was more frequent in patients with MV, and thus, the effect of a previously survived COVID-19-infection might be overestimated regarding protection against a new severe COVID-19-infection. Nevertheless, a post-COVID-status was associated with lower in-hospital mortality. In accordance with the literature, the majority of ventilated patients were of male sex (approximately 70%), obese, and suffered mostly from liver or cardiovascular disease [6,22,23,24]. COVID-19 patients with MV in Germany had a higher risk of in-hospital death than non-ventilated patients (37.0% vs. 16.5%, multivariate regression analysis: OR 3.82), which is in the range of previously published studies (24.5–60.4%) [5,6,23,25].

In our analysis, the in-hospital case-fatality increased with age, which has also been described before [6,25,26,27]. Hospitalized COVID-19 patients in Germany aged 70 years or older died substantially more often than younger patients (28.8% vs. 5.5%, multivariate regression analysis: OR 5.91). Consistent with current literature, heart failure, coronary artery disease, chronic pulmonary disease, liver disease, renal disease, and cancer [27], but VTE and major bleeding events were also identified as significant risk factors for mortality. These results support the concept that patients with already existing cardiovascular disease might be more prone to aggravated and poor outcomes. As expected, the manifestation of the respiratory infection was a strong predictor of in-hospital case-fatality since pneumonia and ARDS were both independently associated with an unfavorable course of illness. Consequently, the use of ECMO, as an escalation of invasive ventilation treatment, was associated with very high in-hospital mortality, as well. Data from the early pandemic period in France suggested an ECMO-case-fatality comparable to those of regular ARDS patients, as opposed to Germany [6].

Consistent with another large study using age-specific COVID-19-associated death data from 45 countries [26], our data reveal low in-hospital mortality of children in their first decade of life, but a frightful increase with every age decade.

With respect to resource availability and allocation, the present study demonstrated large regional differences regarding the hospitalization and in-hospital case-fatality in Germany. These regional discrepancies are of outstanding interest for optimal healthcare management and planning. Furthermore, our study indicates a substantial difference regarding the in-hospital case-fatality between hospitals of rural versus urban areas. COVID-19 patients admitted to hospitals in urban areas had a slightly lower in-hospital mortality than those in rural areas (18.8% vs. 17.5%), although patients with critical illness were mostly transferred from rural to urban hospitals for therapy escalation. These results strengthen the outstanding meaning of those hospitals offering maximal treatment capacities in contrast to regional hospitals, in order to deliver more advanced (respiratory) treatment.

The present study has certain limitations that need consideration. First, as our results are based on administrative and retrospective data, we cannot exclude misclassification or inconsistencies. Additionally, this analysis of the German nationwide inpatient sample was not prespecified; therefore, our findings can only be considered to be hypothesis-generating. Second, patients with confirmed COVID-19 infection, who died out of hospital or were diagnosed post mortem, were not included in the German nationwide inpatient sample. Third, the German nationwide inpatient sample does not report long-term outcomes after the discharge from hospital.

## 6. Conclusions

Our findings demonstrated considerable seasonal and regional differences in the number of patients with COVID-19 and in need of MV. We identified important risk factors for MV, healthcare resource-binding complications, as well as in-hospital fatality. These findings may help to ensure a flexible allocation of intensive care resources, in order to deliver sufficient healthcare capacity at the right time-point to meet the enormous healthcare challenges in the future.

## Figures and Tables

**Table 1:** Patients’ characteristics, medical history, presentation, and adverse in-hospital events of the 176,137 hospitalized patients with confirmed COVID-19 infection in Germany in the year 2020 stratified for in-hospital survival.

Parameters	Survivors (*n* = 144,530; 82.1%)	Non-Survivors (*n* = 31,607; 17.9%)	*p*-Value
Age	67.0 (52.0/80.0)	82.0 (76.0/87.0)	<0.001
Age ≥ 70 years	67,207 (46.5%)	27,122 (85.8%)	<0.001
Female sex	70,693 (48.9%)	13,256 (41.9%)	<0.001
In-hospital stay (days)	8.0 (4.0/14.0)	8.0 (4.0/16.0)	<0.001
**Cardiovascular risk factors**			
Obesity	7798 (5.4%)	1585 (5.0%)	0.006
Diabetes mellitus	34,241 (23.7%)	10,991 (34.8%)	<0.001
Essential arterial hypertension	66,191 (45.8%)	16,289 (51.5%)	<0.001
Hyperlipidaemia	22,205 (15.4%)	5368 (17.0%)	<0.001
**Comorbidities**			
Coronary artery disease	18,356 (12.7%)	7218 (22.8%)	<0.001
Heart failure	17,400 (12.0%)	9719 (30.7%)	<0.001
Peripheral artery disease	3834 (2.7%)	1806 (5.7%)	<0.001
Atrial fibrillation/flutter	23,214 (16.1%)	10,946 (34.6%)	<0.001
Chronic obstructive pulmonary disease	8865 (6.1%)	3289 (10.4%)	<0.001
Chronic renal insufficiency (glomerular filtration rate < 60 mL/min/1.73 m^2^)	17,976 (12.4%)	9396 (29.7%)	<0.001
Cancer	6405 (4.4%)	2596 (8.2%)	<0.001
Severe liver disease	2167 (1.5%)	1972 (6.2%)	<0.001
Charlson comorbidity index	3.0 (1.0/5.0)	6.0 (5.0/8.0)	<0.001
**Respiratory manifestations of COVID-19**			
Pneumonia	80,042 (55.4%)	26,871 (85.0%)	<0.001
Acute respiratory distress syndrome	5990 (4.1%)	5604 (17.7%)	<0.001
**Treatment**			
Intensive care unit	16,662 (11.5%)	10,391 (32.9%)	<0.001
Mechanical ventilation	7655 (5.3%)	4487 (14.2%)	<0.001
Extracorporeal membrane oxygenation (ECMO)	445 (0.3%)	1009 (3.2%)	<0.001
Dialysis	2388 (1.7%)	3187 (10.1%)	<0.001
**Adverse events during hospitalization**			
Cardio-pulmonary resuscitation	551 (0.4%)	2308 (7.3%)	<0.001
Venous thromboembolism	3655 (2.5%)	1332 (4.2%)	<0.001
Acute kidney failure	10,911 (7.5%)	11,164 (35.3%)	<0.001
Myocarditis	171 (0.1%)	55 (0.2%)	0.012
Myocardial infarction	1677 (1.2%)	1076 (3.4%)	<0.001
Stroke (ischaemic or haemorrhagic)	2179 (1.5%)	1017 (3.2%)	<0.001
Intracerebral bleeding	301 (0.2%)	275 (0.9%)	<0.001
Gastro-intestinal bleeding	1802 (1.2%)	1146 (3.6%)	<0.001
Transfusion of blood constituents	7895 (5.5%)	5979 (18.9%)	<0.001

**Table 2:** Patients’ characteristics, medical history, presentation, and adverse in-hospital events of the 176,137 hospitalized patients with confirmed COVID-19 infection in Germany in the year 2020 stratified for the use of mechanical ventilation.

Parameters	No Mechanical Ventilation (*n* = 163,995; 93.1%)	Mechanical Ventilation (*n* = 12,142; 6.9%)	*p*-Value
Age	72.0 (55.0/82.0)	70.0 (59.0/79.0)	<0.001
Age ≥ 70 years	88,192 (53.8%)	6137 (50.5%)	<0.001
Female sex	79,997 (48.8%)	3952 (32.5%)	<0.001
In-hospital stay (days)	7.0 (3.0/13.0)	17.0 (10.0/29.0)	<0.001
**Cardiovascular risk factors**			
Obesity	7945 (4.8%)	1438 (11.8%)	<0.001
Diabetes mellitus	40,795 (24.9%)	4437 (36.5%)	<0.001
Essential arterial hypertension	75,850 (46.3%)	6630 (54.6%)	<0.001
Hyperlipidaemia	25,470 (15.5%)	2103 (17.3%)	<0.001
**Comorbidities**			
Coronary artery disease	23,366 (14.2%)	2208 (18.2%)	<0.001
Heart failure	24,316 (14.8%)	2803 (23.1%)	<0.001
Peripheral artery disease	5122 (3.1%)	518 (4.3%)	<0.001
Atrial fibrillation/flutter	30,877 (18.8%)	3283 (27.0%)	<0.001
Chronic obstructive pulmonary disease	10,926 (6.7%)	1228 (10.1%)	<0.001
Chronic renal insufficiency (glomerular filtration rate < 60 mL/min/1.73 m^2^)	25,373 (15.5%)	1999 (16.5%)	0.004
Cancer	8331 (5.1%)	670 (5.5%)	0.036
Severe liver disease	3206 (2.0%)	933 (7.7%)	<0.001
Charlson comorbidity index	4.0 (2.0/6.0)	5.0 (3.0/7.0)	<0.001
**Respiratory manifestations of COVID-19**			
Pneumonia	95,918 (58.5%)	10,995 (90.6%)	<0.001
Acute respiratory distress syndrome	6847 (4.2%)	4747 (39.1%)	<0.001
**Treatment**			
Intensive care unit	17,631 (10.8%)	9422 (77.6%)	<0.001
Extracorporeal membrane oxygenation (ECMO)	677 (0.4%)	777 (6.4%)	<0.001
Dialysis	3699 (2.3%)	1876 (15.5%)	<0.001
**Adverse events during hospitalization**			
In-hospital case fatality	27,120 (16.5%)	4487 (37.0%)	<0.001
Cardio-pulmonary resuscitation	2107 (1.3%)	752 (6.2%)	<0.001
Venous thromboembolism	3993 (2.4%)	994 (8.2%)	<0.001
Acute kidney failure	17,688 (10.8%)	4387 (36.1%)	<0.001
Myocarditis	181 (0.1%)	45 (0.4%)	<0.001
Myocardial infarction	2425 (1.5%)	328 (2.7%)	<0.001
Stroke (ischaemic or haemorrhagic)	2813 (1.7%)	383 (3.2%)	<0.001
Intracerebral bleeding	437 (0.3%)	139 (1.1%)	<0.001
Gastro-intestinal bleeding	2559 (1.6%)	389 (3.2%)	<0.001

## Data Availability

All code used in this study is publicly available online. The data used in this study are sensitive due to individual patient-level data and will not be made publicly available. The data is available at the Federal Statistical Office of Germany (Statistisches Bundesamt, DEStatis) (source: RDC of the Federal Statistical Office and the Statistical Offices of the federal states, DRG Statistics 2020, and own calculations).

## Supplementary Materials

The following are available online at https://www.mdpi.com/article/10.3390/v14020275/s1, Figure S1. Study flow chart, Figure S2. Rates of in-hospital mortality in hospitalised patients with COVID-19-infection stratified for severity of ARDS, Figure S3. Regional trends regarding total numbers of hospitalised patients with COVID-19-infection in Germany 2020, Table S1: Used ICD and OPS codes for the present analysis.
