# Supplementary material for: Trends and Risk Factors of In-Hospital Mortality of Patients with COVID-19 in Germany: Results of a Large Nationwide Inpatient Sample
Source: Viruses. 2022 Jan 28;14(2):275. doi: 10.3390/v14020275 (PMC8880622; doi:10.3390/v14020275)
Supplement: Supplementary file 1 [file viruses-14-00275-s001.zip › viruses-1564839-supplementary.pdf]

Supplementary Materials

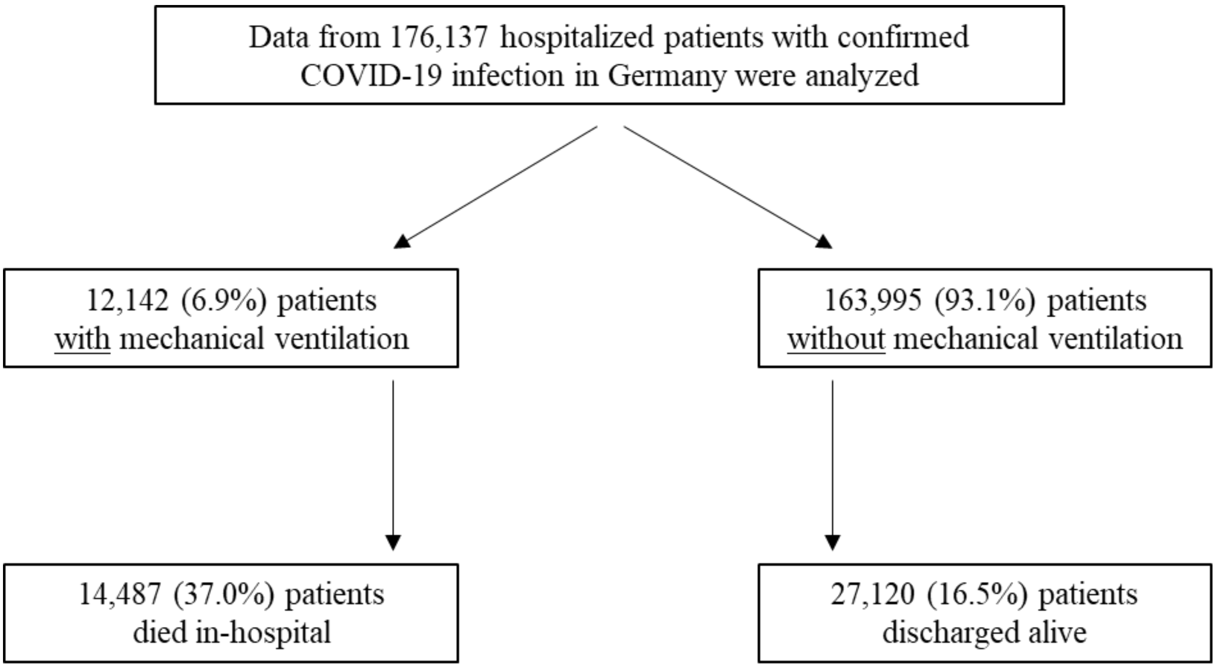

Figure S1. Study flow chart.

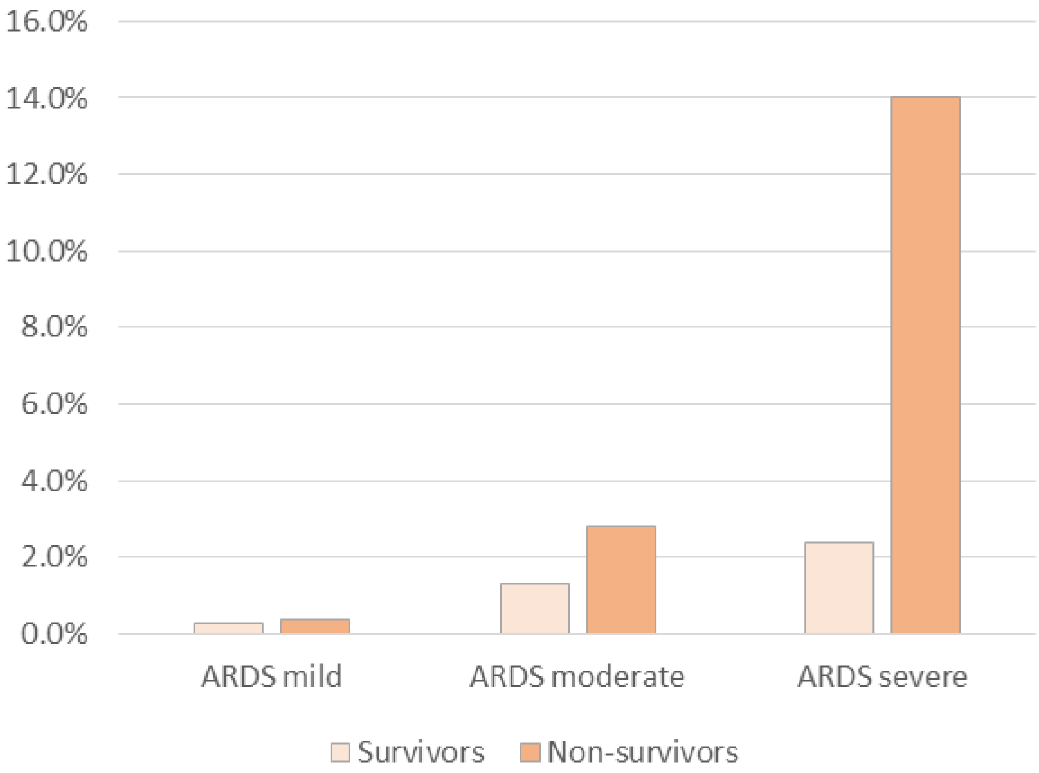

Figure S2. Rates of in-hospital mortality in hospitalised patients with COVID-19-infection stratified for severity of ARDS.

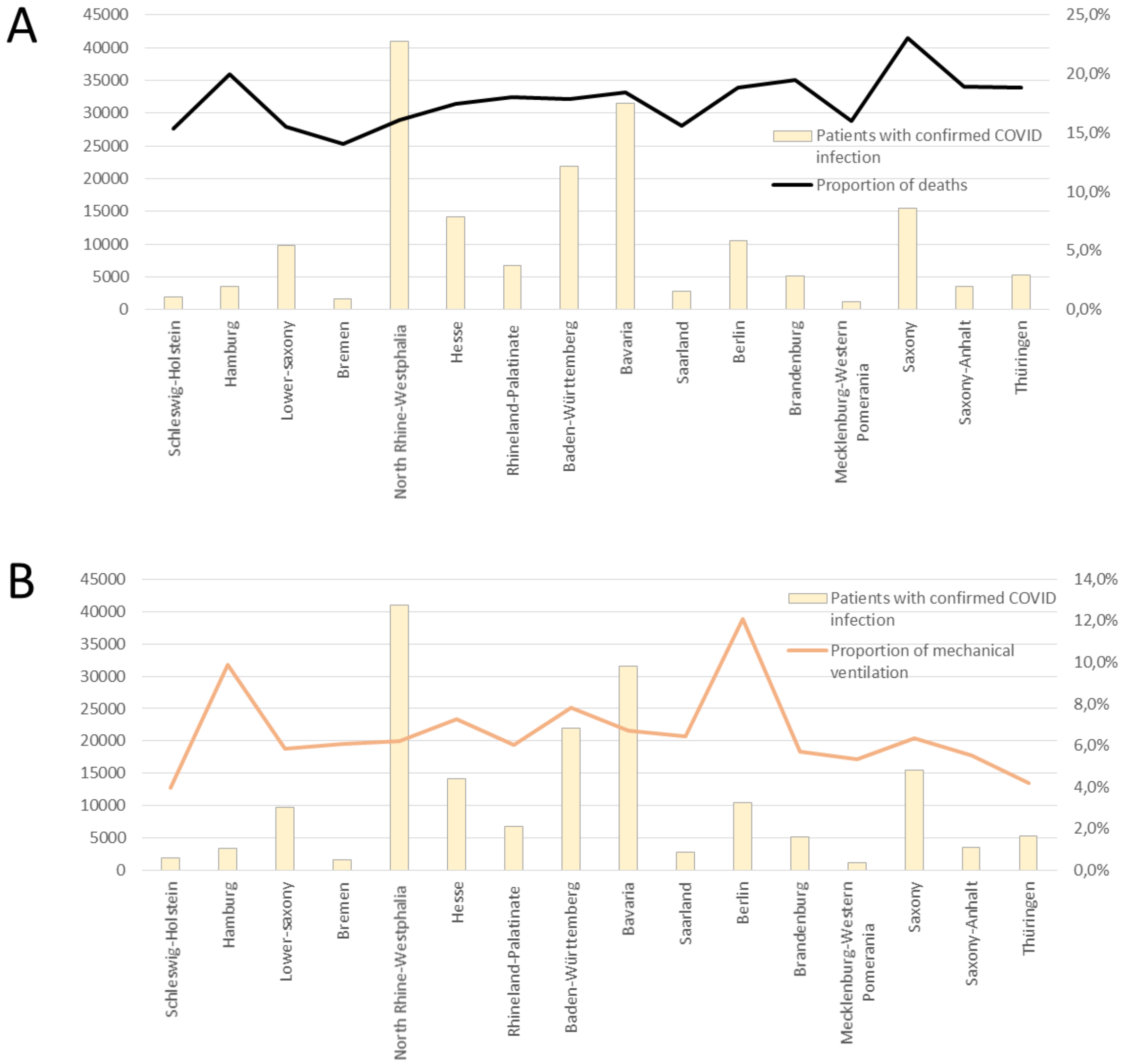

Figure S3. Regional trends regarding total numbers of hospitalised patients with COVID-19-infection in Germany 2020. **Panel A**—Regional trends regarding total numbers of hospitalised patients with COVID-19and proportion of deaths stratified for federal states. **Panel B**—Regional trends regarding total numbers of hospitalised patients with COVID-19-infection as well as COVID-patients and proportion of mechanical ventilations stratified for federal states.

Table S1. Used ICD and OPS codes for the present analysis.

Acute infection of the upper airways (AIOA, ICD code J0.6) | Acute infection of the lower airways (IUA, ICD code J22) | Acute renal failure (ICD code N17) | Acute respiratory distress syndrome (ARDS, ICD code J80) | ARDS mild (ICD code J80.01) | ARDS moderate (ICD code J80.02) | ARDS severe (ICD code I80.03) | Arterial hypertension (ICD code I10) | Atrial fibrillation/flutter (ICD code I48) | Cancer (ICD codes C00-C97) | Confirmed COVID Infection (U07.1) | Chronic obstructive pulmonary disease (ICD code J44) | Coronary artery disease (ICD code I25) | Diabetes mellitus (ICD code E10-E14) | Dialysis (OPS codes 8-853, 8-854) | Extracorporeal Membrane Oxygenation (ECMO, OPS code 8-852) | Gastro-intestinal bleeding (ICD code K92.0-K92.2) | Heart failure (ICD code I50) | Hyperlipidemia (ICD code E78) | Intensive care unit (ICU, OPS codes 8-980, 8-98d and 8-98f) | Intracerebral bleeding (ICB, ICD code I61) | Mechanical ventilation (OPS codes 8-71) | Myocarditis (ICD code I40) | Myocardial infarction (ICD codes I21-I22) | Obesity (ICD code E66) | Peripheral artery disease (ICD code I70.2) | Pneumonia (ICD codes J12-J18) | POST-COVID status (ICD code U09.9, U7.4, U08.9, U0.73) | Renal insufficiency with glomerular filtration rate <60

mL/min/1.73 m2 (ICD codes N18.3, N18.4, N18.5, N18.83, N18.84, N18.0) | Cardio-pulmonary resuscitation (CPR, OPS code 8-77) | Severe liver disease (ICD code K70.2-K70.4, K71-K75, K76.1-K76.7, K77) | Surgery (OPS codes 5-01 to 5-99) | Ischemic or hemorrhagic Stroke (ICD codes I61-I64) | Transfusion of erythrocytes (OPS codes 8-800) | Venous thromboembolism (VTE, ICD codes I26, I80-I82).
